# The genetic profile of B-cell acute lymphoblastic leukaemia diagnosed in Pretoria, South Africa: A 10-year retrospective study

**DOI:** 10.4102/jcmsa.v4i1.432

**Published:** 2026-10-13

**Authors:** Kirsten Olivier, Annelli Marais, Keryn Thompson, Amery Badenhorst, Angela Tennant, Joachim Potgieter

**Affiliations:** 1 Department of Haematology, Faculty of Health Sciences, University of Pretoria, Pretoria, South Africa; 2 Department of Haematology, Tshwane Academic Division, National Health Laboratory Services, Pretoria, South Africa

**Keywords:** B-ALL, cytogenetics, FISH, *TCF3::PBX1*, t(1;19), South Africa

## Abstract

**Background:**

B-cell acute lymphoblastic leukaemia (B-ALL) comprises genetically distinct subtypes with important prognostic implications. South African data suggest a different genetic landscape to other geographic regions, with a higher frequency of t(1;19). This study aimed to describe the genetic profile of B-ALL diagnosed at a South African tertiary haematology laboratory over a 10-year period.

**Methods:**

In this retrospective, descriptive study, all B-ALL cases (2014–2023) recorded in flow cytometry and cancer registries were identified. Demographic and laboratory data, including immunophenotypic and genetic findings, were extracted from the laboratory information system. Genetic work-up included karyotyping, a routine fluorescence in situ hybridisation panel: *KMT2A* rearrangement, t(1;19), t(12;21) and t(9;22), and real-time quantitative polymerase chain reaction where available.

**Results:**

A total of 173 patients were included (54.9% male; 75.7% < 19 years). Genetic testing was requested in 146 cases (84.4%), with definitive results in 115 (78.8%). Among informative cases, t(1;19) was most frequent (26.1%), followed by t(9;22) (12.2%), t(12;21) (10.4%), high hyperdiploidy (9.6%), *KMT2A* rearrangement (7.0%) and hypodiploidy (0.9%). The t(1;19) translocation predominated in paediatric patients, while t(9;22) was more common in adults. Both t(12;21) and *KMT2A* rearrangements were restricted to patients < 19 years.

**Conclusion:**

This cohort demonstrates a high frequency of t(1;19) and age-specific variation in B-ALL genetic subtypes, suggesting a distinct regional genetic profile. Expanded diagnostic testing and multicentre African studies are needed to refine risk stratification and guide context-appropriate care.

**Contribution:**

This study provides data on the distribution of selected genetic subtypes of B-ALL in a South African population, identifying differences in subtype frequency and age distribution that may inform regional diagnostic strategies and risk-directed care.

## Introduction

Acute lymphoblastic leukaemia (ALL) is a haematological malignancy arising from the neoplastic proliferation of immature lymphocytes in the bone marrow. It can be of either B- or T-cell lineage, with B-cell acute lymphoblastic leukaemia (B-ALL) accounting for approximately 75% of cases.^1^ B-cell acute lymphoblastic leukaemia is most commonly diagnosed in childhood, with a second, smaller incidence peak in adults around the age of 50 years.^2^ The incidence appears to be higher in males than females.^3,4,5^

A number of distinct genetic subtypes of B-ALL have been described, each with a unique pattern of clinical and prognostic features.^6^ Recurrent chromosomal aberrations include whole-chromosome aneuploidy and structural abnormalities such as translocations that generate chimeric fusion genes or deregulate oncogenes.^6,7,8^ Detection of these initiating chromosomal aberrations is important for diagnosis, risk classification and, for certain subtypes, targeted therapy.^7^ An overview of the common cytogenetic subtypes is given in Table 1.

**TABLE 1:** Description and prognosis of selected genetic subtypes in B-cell acute lymphoblastic leukaemia.

Genetic subtype	Description	Category	Prognosis
High hyperdiploidy	> 50 chromosomes	Aneuploid	Excellent
Hypodiploidy	< 44 chromosomes	Aneuploid	Poor
*ETV6*::*RUNX1*	t(12;21)(p13;q22)	TF rearrangement	Excellent in children, undetermined in adults
*KMT2A*-r	t(v;11q23)	TF rearrangement	Poor
*TCF3*::*PBX1*	t(1;19)(q23;p13)	TF rearrangement	Intermediate to good; CNS relapse
*BCR*::*ABL1*	t(9;22)(q34;q11.2)	Kinase driven	Poor; improved with TKI

*Source*: Zhang X, Rastogi P, Shah B, Zhang L. B lymphoblastic leukaemia/lymphoma: New insights into genetics, molecular aberrations, subclassification and targeted therapy. Oncotarget. 2017;8(39):66728–66741. https://doi.org/10.18632/oncotarget.19271^8^; Roberts KG. Genetics and prognosis of ALL in children vs adults. Hematology Am Soc Hematol Educ Program. 2018;2018(1):137–145. https://doi.org/10.1182/asheducation-2018.1.137^9^

TF, transcription factor; *KMT2A*-r, *KMT2A* rearranged; TKI, tyrosine kinase inhibitor; CNS, central nervous system.

The frequencies of these genetic subtypes vary geographically. In paediatric cohorts from Europe and the United States, t(12;21)/*ETV6::RUNX1* is the most commonly reported genetic aberration.^9,10^ By contrast, both t(12;21) and high hyperdiploidy appear at lower frequencies in several Middle Eastern and Asian series, while t(9;22)/*BCR::ABL1* is reported more often among Chinese and Malaysian patients than in other population groups.^11,12,13,14,15^

To date, African data on the distribution of B-ALL genetic subtypes remain limited. In Johannesburg, Vaughan et al. identified t(1;19)/*TCF3::PBX1* as the most frequent recurrent aberration in B-ALL,^10^ a pattern echoed in a paediatric ALL cohort from Polokwane.^16^ The translocation t(1;19) has also been reported to occur more frequently among African American children with B-ALL, suggesting an ethnic or ancestry-related predisposition to this chromosomal rearrangement.^17,18^ Ethnic variability in subtype frequencies is further supported by Malaysia–Singapore paediatric data.^15^

With regional data remaining sparse, we describe a comprehensive 10-year genetic profile of B-ALL diagnosed at a tertiary haematology laboratory in Pretoria to better define the South African landscape.

## Research methods and design

### Study design and setting

This was a retrospective, descriptive, cross-sectional study including all B-ALL cases diagnosed at the National Health Laboratory Service (NHLS) – Tshwane Academic Division (Pretoria, South Africa) between 01 January 2014 and 31 December 2023. The laboratory provides diagnostic services (flow cytometry and bone marrow morphology) for public-sector hospitals in Gauteng North, Gauteng East and the Mpumalanga, North West and Limpopo provinces.

### Case identification and inclusion criteria

Cases were identified through the laboratory’s cancer and flow cytometry registries. All newly diagnosed cases of B-ALL confirmed by flow cytometry were included in the study. B-cell acute lymphoblastic leukaemia was diagnosed by multiparameter flow cytometry demonstrating a precursor B-cell immunophenotype, characterised by expression of B-cell lineage-associated markers, including CD19, CD22 and CD79a, together with blast-associated markers such as TdT and CD34, and variable expression of CD10. Findings were interpreted in conjunction with morphology and available clinical information. Cases without confirmatory flow cytometry were excluded. For the genetic analysis, only cases with informative genetic results were included; cases without genetic testing and those with non-informative genetic results were excluded.

### Data collection and variables

From the laboratory information system (TrakCare, InterSystems, Cambridge, Massachusetts, United States [US]), we extracted demographics (age, sex), peripheral blood counts (haemoglobin, white cell count and platelet count), peripheral blood and bone marrow blast percentages, immunophenotype, karyotype, fluorescence in situ hybridisation (FISH) results and real-time quantitative polymerase chain reaction (RT-qPCR) results, where available. Karyotyping, FISH and RT-qPCR were performed at accredited regional laboratories. The range of FISH investigations offered did not change over the 10-year study period. The standard B-ALL FISH panel comprised probes for *KMT2A* rearrangement (*KMT2A*-r), *TCF3::PBX1, ETV6::RUNX1* and *BCR::ABL1*. Testing was requested at the discretion of the treating clinician according to standard laboratory practice and was not routinely modified according to patient age. Real-time quantitative polymerase chain reaction testing was restricted to *BCR::ABL1*-positive cases. Real-time quantitative polymerase chain reaction results were not consistently available because molecular testing was performed selectively according to clinical practice and was not available for all *BCR::ABL1*-positive cases during the study period.

### Data analysis

Descriptive statistics were used to summarise the demographic, laboratory and genetic characteristics of this retrospective cohort. Categorical variables are presented as frequencies and percentages, while continuous variables are presented as mean (standard deviation [s.d.]) or median (interquartile range [IQR]), as appropriate. As this study was designed to characterise the distribution of genetic subtypes rather than evaluate associations or test predefined hypotheses, inferential statistical analyses, including confidence intervals, subgroup comparisons and adjustments for multiple comparisons, were not undertaken. Data analysis was performed using Microsoft Excel and Statistica software, Version 14.0.1.25 (TIBCO Software, Palo Alto, California, US).

### Ethical considerations

Ethical clearance to conduct this study was obtained from Faculty of Health Sciences Research Ethics Committee, University of Pretoria (No: 274/2024-Line 1). Permission to use laboratory data was obtained from the NHLS Academic Affairs and Research Office (Ref. PR2455013). De-identified data were stored in a secure database; no direct patient contact occurred. The study adhered to the principles of the Declaration of Helsinki.

## Results

### Study population

A total of 173 patients with B-ALL were identified over the 10-year period. B-cell acute lymphoblastic leukaemia was slightly more prevalent in males than females (54.9% vs 45.1%), and more than 50% of cases occurred in children under 10 years of age. Approximately 76% of cases occurred in patients under 19 years of age (children and adolescents). Demographic data are presented in Table 2, laboratory findings at diagnosis are summarised in Table 3, and genetic testing uptake and yield are depicted in Figure 1.

**FIGURE 1:**
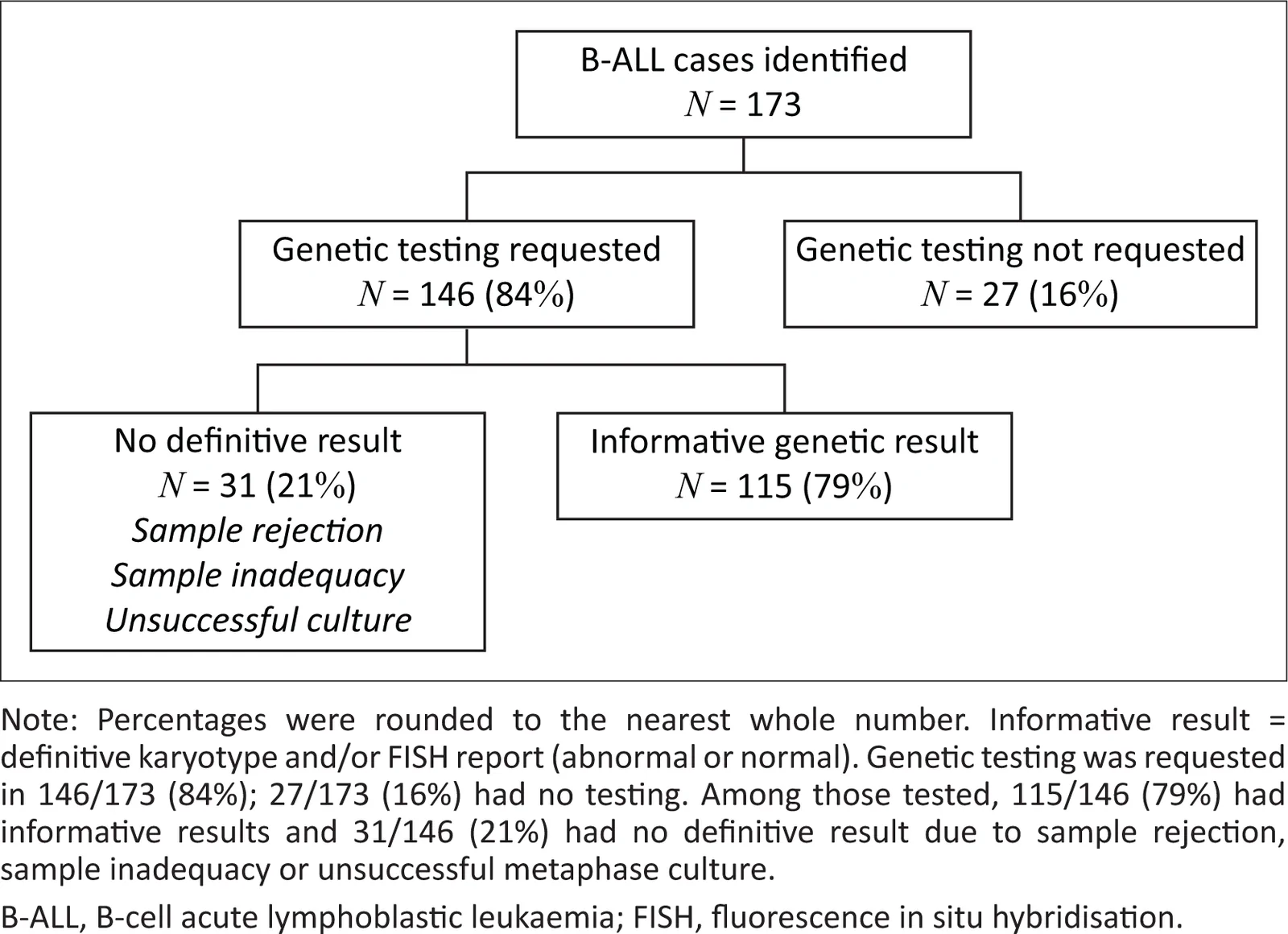
Flow of genetic testing and yield among B-cell acute lymphoblastic leukaemia cases (*N* = 173).

**TABLE 2:** Demographic characteristics of the B-cell acute lymphoblastic leukaemia cohort (*N* = 173).

Variable	*n*	%
**Sex**		
Male	95	54.9
Female	78	45.1
**Age (years)**		
< 10	87	50.3
10–18	44	25.4
19–39	27	15.6
≥ 40	15	8.7

**TABLE 3:** Laboratory characteristics of the B-cell acute lymphoblastic leukaemia cohort at diagnosis (*N* = 173).

Variable	Value		Dispersion	
	Mean	Median	s.d.	IQR
Haemoglobin (g/dL)	7.8	-	2.65	-
White cell count (×10^9^/L)	-	12.95	-	4.60–54.04
Platelet count (×10^9^/L)	-	36	-	15–86
Peripheral blood blasts (%)	-	61	-	34.5–82.5
Bone marrow blasts (%)	-	83	-	68–91

IQR, interquartile range; s.d., standard deviation.

### Distribution of recurrent genetic aberrations

Among the informative cases (*n* = 115), t(1;19) was the most common genetic aberration detected, occurring in 30/115 (26.1%) of all cases and found in almost one third of patients younger than 19 years of age (28/88, 31.8%). Only 2/30 (6.7%) of t(1;19)-positive cases occurred in adult patients. The t(12;21) and *KMT2A*-r were seen exclusively in children and adolescents. The most prevalent recurrent genetic abnormality seen in adult patients was t(9;22). The overall distribution of cytogenetic subtypes is summarised in Figure 2. Figure 3 contrasts age-specific patterns (children and adolescents versus adults).

**FIGURE 2:**
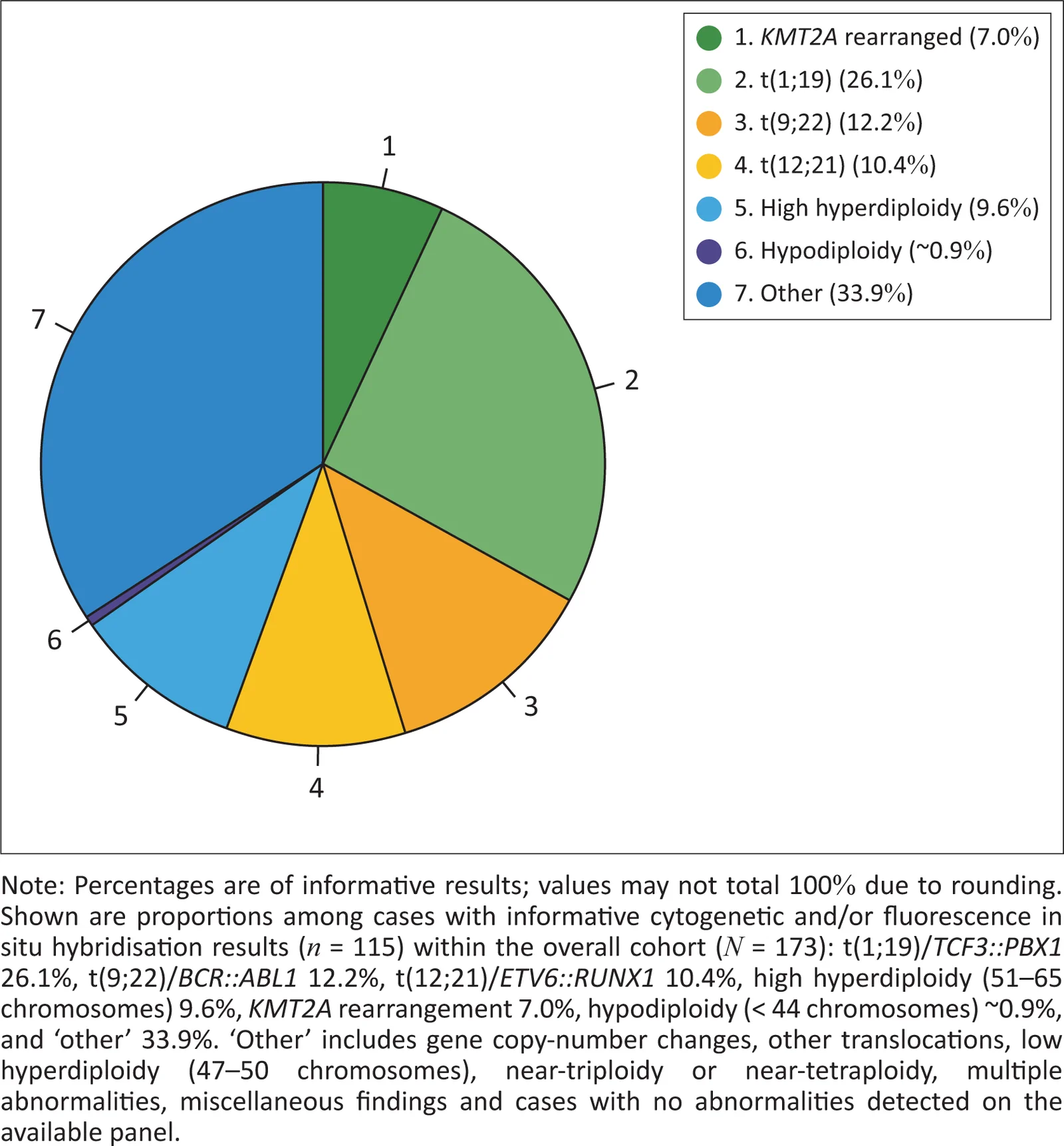
Distribution of recurrent genetic abnormalities in B-cell acute lymphoblastic leukaemia.

**FIGURE 3:**
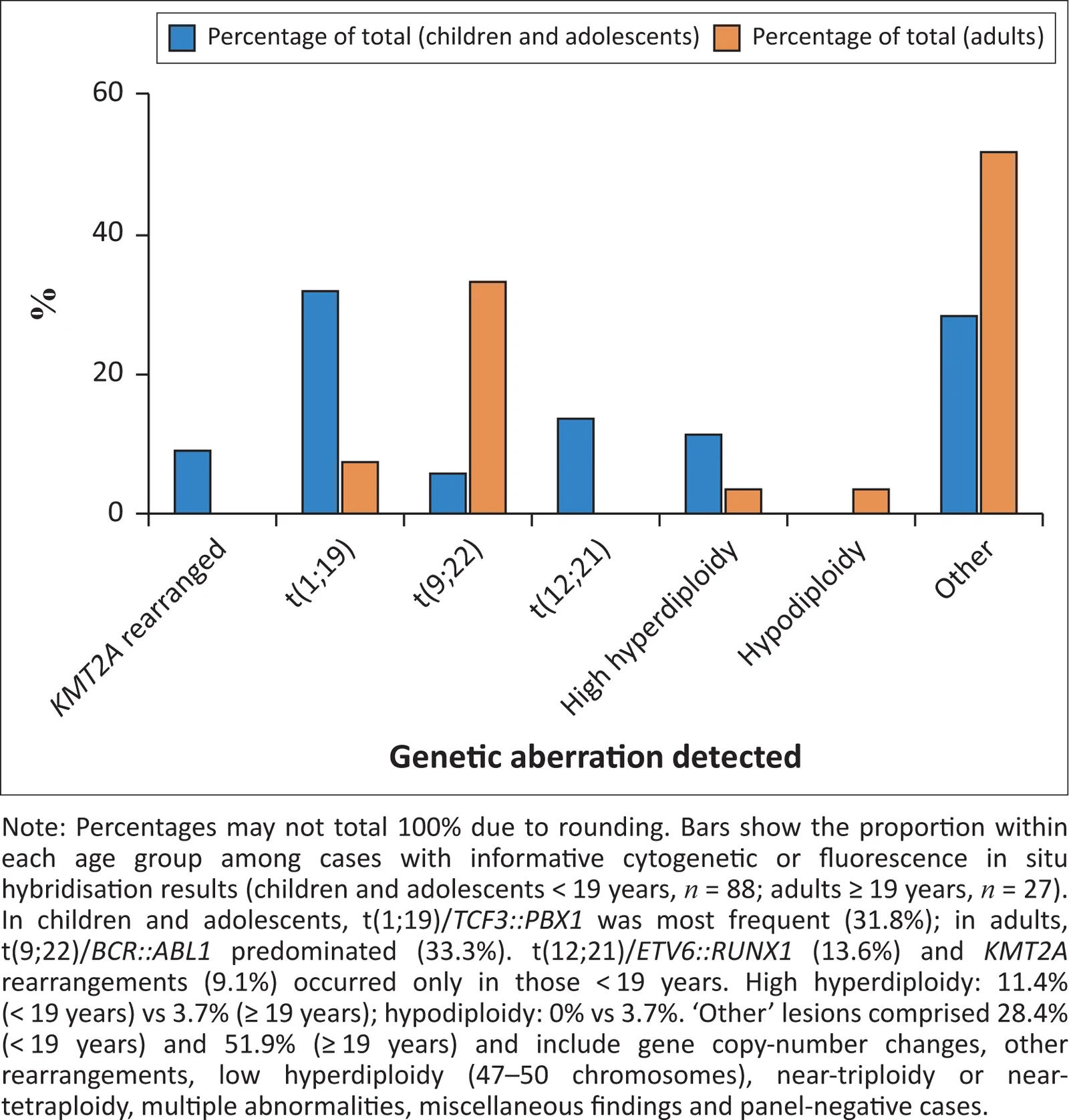
Age-stratified distribution of recurrent genetic abnormalities in B-cell acute lymphoblastic leukaemia.

### Karyotypic context of recurrent genetic subtypes

t(1;19)/*TCF3::PBX1* – Of the cases with karyotypes, 17/23 (73.9%) had additional abnormalities. The rearrangement was unbalanced in 23/30 cases (76.7%) (der(19)t(1;19)); 1 variant showed both balanced and unbalanced clones; 1 ‘jumping’ 1q translocation was observed. The 1q gain was common (partial trisomy in 13/17 and tetrasomy in 2/17 unbalanced cases with karyotypes). Modal chromosome numbers were usually 46.

t(9;22)/*BCR::ABL1* – Karyotypes were available for 5/14 (35.7%) of cases. All five cases with karyotypes had additional lesions (including other translocations, der(22), +8, and –9). Double Philadelphia chromosome (Ph) was documented in two adults (one evolved from chronic myeloid leukaemia [CML]). Real-time quantitative polymerase chain reaction (*n* = 4): two p190 and two p210 transcripts (the latter including the CML transformation).

t(12;21)/*ETV6::RUNX1* – Cryptic on karyotype in all evaluated cases. Frequent co-lesions involved 12p13 (deletions, additions or translocations), del(6q) and aneuploidy (gains of 10, 21; losses of 9, 12, X).

*KMT2A* rearrangements – Partner chromosomes included 4, 9, 19 and 17. Additional abnormalities were uncommon and were limited to trisomy 6 or trisomy 21.

High hyperdiploidy – Universal +21; tetrasomy 21 in 7/9 informative karyotypes. Other recurrent gains: 10, 14, 18, X, 6, 4, 17.

Hypodiploidy – Single low-hypodiploid case with broad losses (2–5, 7, 12–18, 20).

Other – 39 cases were classified as ‘other’. These comprised gene copy-number changes (*n* = 17), other rearrangements (*n* = 5), low hyperdiploidy (47–50 chromosomes, *n* = 5), near-triploidy or near-tetraploidy (*n* = 3), multiple abnormalities (*n* = 3), miscellaneous (*n* = 1) and no abnormalities detected by karyotyping or FISH (*n* = 5).

## Discussion

In this 10-year, single-laboratory cohort, t(1;19)/*TCF3::PBX1* emerged as the most frequent recurrent aberration (26.1% overall; 31.8% in children and adolescents), mirroring prior South African data and contrasting with the ≤ 5% reported in many high-income settings.^9,10,19^ Taken together with the higher t(1;19) frequencies reported among patients of African descent in US datasets,^17,18^ these findings are consistent with previous observations suggesting a possible ancestry-related contribution to t(1;19) frequency; however, no genomic ancestry data were available in the present study, and this hypothesis requires further investigation. The predominance of an unbalanced translocation in our cohort, der(19)t(1;19) in 76.7%, aligns with the recognised biology of this subtype.^20^ Additional cytogenetic aberrations were common (73.9%), but most karyotypes had a modal number of 46 chromosomes.

t(9;22)/*BCR::ABL1* was the second most frequent lesion (12.2% overall) and, in alignment with previous studies, predominated in adults (33.3%).^8^ Co-lesions were ubiquitous in the karyotyped subset (including other translocations, derivative 22, +8 and −9), and double Ph was documented in two adults (including one CML blast transformation), consistent with genomic evolution in *BCR::ABL1*-positive disease. Real-time quantitative polymerase chain reaction showed both p190 and p210 transcripts, underscoring the need for routine transcript typing to guide measurable residual disease (MRD) strategies and tyrosine kinase inhibitor (TKI) selection. The adult enrichment echoes international findings,^21^ while the lower paediatric prevalence in our cohort aligns with UK data and remains below that reported in parts of Southeast Asia.^15,19^

*ETV6::RUNX1*/t(12;21) accounted for 10.4% overall and was restricted to patients under 19 years of age (predominantly < 10 years of age). As anticipated, all evaluable cases were cryptic on karyotype, reinforcing the value of targeted FISH and ribonucleic acid (RNA) methods. Recurrent 12p13 lesions (deletions, additions or translocations), del(6q) and aneuploidy accompanied many cases, reflecting known cooperating events that do not typically alter the favourable prognosis associated with this subtype.^22^

*KMT2A*-rearranged B-ALL comprised 7.0% of informative results and occurred exclusively in children and adolescents. *KMT2A* rearrangements are reported to occur frequently in infants diagnosed with B-ALL, accounting for at least 67% of infant cases of ALL.^18^ In our cohort, of cases for which an informative genetic result was obtained, only a single infant (< 1 year of age) was identified; this patient tested positive for *KMT2A*-r. The prevalence of *KMT2A*-r in non-infant children and adolescents in our cohort was 8.0% (7/87 cases); international studies have reported a prevalence of 1%–2% in older children.^8,9^ The somewhat higher proportion observed in our cohort could reflect referral or testing patterns or small-number effects; broader, multicentre sampling would clarify whether this represents a true population signal.

Partner loci included chromosomes 4, 9, 17 and 19. Co-abnormalities were infrequent and limited to aneuploidy (one case with trisomy 6 and one with trisomy 21) in this series.

High hyperdiploidy (51–65 chromosomes) was relatively uncommon (9.6% overall; 11.4% in children and adolescents) and occurred at a lower frequency than reported in high-income paediatric series.^19^ All informative karyotypes showed chromosome 21 gain (with tetrasomy 21 dominant), and frequent gains of 10, 14, 18, X, 6, 4 and 17, consistent with canonical patterns.^23^ It should be highlighted that some ‘other’ cases had copy-number alterations without metaphase karyotypes, raising the possibility of misclassification and underestimation of high-hyperdiploid disease in this series.

Hypodiploidy was rare (one low-hypodiploid adult), but the true prevalence may be under-ascertained where karyotyping was unavailable, as targeted FISH has limited ability to detect whole-genome ploidy changes.

The broad ‘other’ group (33.9%) encompassed gene copy-number alterations, additional rearrangements, low hyperdiploidy (47–50), near-triploidy or near-tetraploidy and panel-negative findings. The routine diagnostic panel used during the study period was designed to detect a limited number of recurrent cytogenetic abnormalities and therefore does not fully capture the molecular diversity of B-ALL recognised in contemporary classification systems. Recently described entities, including Ph-like B-ALL, *DUX4*-rearranged B-ALL, *ZNF384*-rearranged B-ALL, *MEF2D*-rearranged B-ALL, *CRLF2*-altered B-ALL and intrachromosomal amplification of chromosome 21 (iAMP21), were not systematically assessed and may account for a proportion of cases classified as ‘other’. Expanded genomic approaches, including chromosomal microarray analysis, targeted RNA sequencing and next-generation sequencing-based assays, would permit more comprehensive subclassification and may alter the observed distribution of genetic subtypes in this population.

### Strengths and limitations

Strengths include the decade-long study period, sizeable cohort and consistent laboratory workflows. Limitations include the retrospective single-centre design, the predominance of public-sector referrals, incomplete genetic characterisation resulting from both untested cases and non-informative results, reliance on send-out cytogenetic testing and the restricted routine FISH panel, which did not systematically assess recently recognised genomic subtypes such as Ph-like ALL, *DUX4*-, *ZNF384*- and *MEF2D*-rearranged B-ALL or iAMP21. In this resource-constrained setting, incomplete testing reflected clinician-directed requesting practices, referral constraints, sample inadequacy and culture failure. Consequently, patients who underwent complete genetic testing may have differed systematically from those who were not tested with respect to disease severity, referral patterns or clinician requesting practices, while unsuccessful testing may have disproportionately affected certain genetic subtypes, introducing selection bias and potentially influencing the observed subtype frequencies. Furthermore, some cases classified as ‘other’ may represent entities identifiable using expanded genomic testing, and hyperdiploidy and hypodiploidy may have been underestimated. Finally, treatment response, MRD, relapse and survival data were not collected, precluding assessment of the prognostic significance of the observed genetic abnormalities.

### Implications and future directions

The prominence of t(1;19) and adult *BCR::ABL1* positivity has direct diagnostic and therapeutic implications in our setting. Knowledge of the local frequency of *TCF3::PBX1* and *BCR::ABL1* may assist laboratories in prioritising limited molecular resources and designing context-appropriate diagnostic algorithms. Recommendations include baseline testing for *TCF3::PBX1* and *BCR::ABL1*, with reflex p190/p210 typing in *BCR::ABL1*-positive cases to direct TKI use and transcript-matched MRD; maintaining FISH coverage for *ETV6::RUNX1* and *KMT2A*-r in children; and expanding access to copy-number arrays or targeted RNA sequencing to capture Ph-like lesions and iAMP21. Prospective, multicentre African studies with harmonised, expanded genomic profiling and linked clinical outcomes are needed to refine risk stratification and support context-appropriate, targeted care.

## Conclusion

This 10-year cohort demonstrates a high frequency of t(1;19) and age-linked distributions of key genetic subtypes. These findings are consistent with previous South African reports and suggest regional variation in the genetic landscape of B-ALL that warrants confirmation in larger multicentre studies.
